# Platelet-rich plasma in Achilles tendon healing 2 (PATH-2) trial: statistical analysis plan for a multicentre, double-blinded, parallel-group, placebo-controlled randomised clinical trial

**DOI:** 10.1186/s13063-018-2840-z

**Published:** 2018-08-29

**Authors:** Michael M. Schlüssel, David J. Keene, Susan Wagland, Joseph Alsousou, Sarah E. Lamb, Keith Willett, Susan J. Dutton, on behalf of the PATH-2 Trial Study Group, Keith Willett, Joseph Alsousou, Sarah E. Lamb, Paul Harrison, Philippa Hulley, Scott Parsons, Jacqueline Thompson, Chris Byrne, Lesley Morgan, Emma Roberts, Daryl Hagan, Ramona Barbu, Michael Schlüssel, Susan Dutton, Roger Smith, Catriona Graham, Dylan Morrissey, John O’Byrne, Gustaaf Reurink, Dishan Singh, Sarah Webb, Chao Huang, Andy Goldberg, Carey McClellan, Robert C. Handley, Maneesh Bhatia, Andrew Kelly, Steve Hepple, Michael Carmont, Paul Hodgson, Nima Heidari, Jonathan Young, Gareth Stables, Ravindran Ranjith, Simon Frostick, Jim Carmichael, Claire Topliss, Lyndon Mason, Moez Ballal, Nasser Kurdy, Mark Davies, Adrian Hughes, Simon Barnes, Matthew Solan

**Affiliations:** 1 Centre for Statistics in Medicine, Nuffield Department of Orthopaedics, Rheumatology and Musculoskeletal Sciences, University of Oxford, Oxford, UK; 2 Kadoorie Centre for Critical Care Research, John Radcliffe Hospital, Nuffield Department of Orthopaedics, Rheumatology and Musculoskeletal Sciences, University of Oxford, Oxford, UK; 3 Centre for Rehabilitation Research, Nuffield Departments of Orthopaedic Rheumatology and Musculoskeletal Sciences, University of Oxford, Oxford, UK; 4 Institute of Translational Medicine, University of Liverpool, Liverpool, UK; 5 Oxford Clinical Trials Research Unit, Centre for Statistics in Medicine, Nuffield Department of Orthopaedics, Rheumatology and Musculoskeletal Sciences, University of Oxford, Oxford, UK

**Keywords:** Statistical analysis plan, Randomised controlled trial, Platelet-rich plasma, Achilles tendon, Rupture

## Abstract

**Background:**

There has been a recent steep growth in platelet-rich plasma (PRP) use for musculoskeletal conditions, but findings from high quality clinical trial data are lacking in the literature. Here, we describe the statistical analysis plan (SAP) for the Platelet-rich plasma in Achilles Tendon Healing 2 (PATH-2) trial.

**Methods:**

PATH-2 is a pragmatic, parallel-group, multi-centre, double-blinded, randomised, placebo-controlled, superiority trial. The study aims to evaluate the clinical efficacy of PRP in acute Achilles tendon rupture in terms of muscle-tendon function. Patients are identified in the orthopaedic/trauma outpatient clinic. The primary outcome is muscle-tendon work capacity from the Heel Rise Endurance Test result, expressed as the Limb Symmetry Index (work, in joules), at 24 weeks post randomisation. Multivariate linear regression adjusting for the stratification factors (centre and age) and additional prognostic factors will be used to investigate the adjusted effect of the intervention. The analysis will be by modified intention-to-treat. Sensitivity analysis will assess the internal validity of the trial results by performing a per-protocol analysis. Safety will be summarised by treatment arm for all patients who started treatment. Secondary patient-reported outcome measures will be analysed using linear mixed effects models to allow all data collected at all follow-up points to be considered. Missing data will be summarised and reported by treatment arm. Missing data imputation will be performed, if appropriate.

**Discussion:**

The PATH-2 trial will be reported in accordance with the CONSORT statement. This SAP publication will avoid bias arising from prior knowledge of the study results. Any changes or deviations from the current SAP will be described and justified in the final report.

**Trial registration:**

ISRCTN registry: ISRCTN54992179, assigned 12 January 2015. ClinicalTrials.gov: NCT02302664, received 18 November 2014. UK Clinical Research Network Study Portfolio Database: ID 17850.

**Electronic supplementary material:**

The online version of this article (10.1186/s13063-018-2840-z) contains supplementary material, which is available to authorized users.

## Background

Despite the lack of high quality clinical trial data, there has been a recent steep growth in platelet-rich plasma (PRP) use for musculoskeletal conditions given it is of relatively low cost and minimally invasive. In 2015, the European PRP market was valued at over US$ 35 million, and is projected to grow at a Compound Annual Growth Rate of more than 6% between 2016 and 2024 [1]. There is also empirical evidence that PRP injections are being introduced in National Health Service (NHS) clinical practice, as evidenced by the increasing appearance in the media, for its potential application in the treatment of traumatic musculoskeletal injury.

Without evidence of efficacy, the consequences of this increased use range from the NHS incurring extra costs for a treatment with unproven clinical effects to the non-deployment of an effective autologous intervention. A recent meta-analysis of PRP for orthopaedic conditions stated the need for adequately powered studies to investigate the effect of PRP using disease-specific and patient-important outcomes [2]. There is therefore a pressing need to undertake an adequately powered and robustly designed study before the use of PRP becomes widely adopted.

Here, we describe the details of the statistical analysis plan for the Platelet-rich plasma in Achilles Tendon Healing 2 (PATH-2) trial [3].

## Methods

### Study design

PATH-2 is a pragmatic, parallel-group, multi-centre, double-blinded, randomised, placebo-controlled superiority trial. The study aims to evaluate the clinical efficacy of PRP in acute Achilles tendon rupture (ATR) recovery in terms of muscle-tendon function. The PATH-2 study is taking place in 18 NHS hospitals across the UK. Patients are identified in the orthopaedic/trauma outpatient clinic, usually following an emergency hospital attendance for an ATR. Two sub-studies are embedded within the main study to investigate the mechanism of PRP in tendon healing. Additional details on the PATH-2 study design can be found elsewhere [3].

### Trial objectives and endpoints

The primary outcome is muscle-tendon function, as estimated by the limb symmetry index (LSI), a measure obtained with the Heel Rise Endurance Test (HRET), which is a validated objective assessment of calf-muscle work capacity [4]. The HRET involves the participant standing on one leg, and raising and lowering the heel repeatedly until fatigued. The uninjured leg is tested first. Data from the movements are obtained using a computer-controlled linear encoder. The linear encoder measures the height of each heel rise executed and this information is used in conjunction with the participant’s body weight to calculate the work performed during the test. The work during the HRET is measured for each lower limb in joules (J). The performance of each limb is then used to generate the LSI according to the following formula:

$$ \mathrm{LSI}=\frac{\mathrm{Injured}\ \mathrm{limb}\ \mathrm{measurement}}{\mathrm{Uninjured}\ \mathrm{limb}\ \mathrm{measurement}}\times 100 $$

The time window for performing the HRET data is 24 weeks (− 2 or + 8 weeks to allow some flexibility). A summary of the study objectives and endpoints (including the secondary outcomes) is presented in Table 1.

**Table 1:** Study objectives and endpoints

Objectives	Endpoints
Primary: • Evaluate the clinical efficacy of platelet-rich plasma (PRP) in acute Achilles tendon rupture (ATR) recovery in terms of muscle-tendon function	At 24 weeks post treatment: • Limb Symmetry Index of work capacity during heel-rise endurance test (HRET); maximum heel-rise height and repetitions during HRET will be reported as secondary outcomes
Secondary: • Evaluate the clinical efficacy of PRP in acute ATR in terms of participant-reported functional recovery, pain and quality of life	During the first 2 weeks post treatment: • Visual analogue scale pain daily diary [15] At 4, 7, 13 and 24 weeks post treatment: • Achilles Tendon Rupture Score [16] • Patient-Specific Functional Scale [17, 18] • SF-12 (acute) [19]
Exploratory (sub-studies): • Determine the key components of PRP that contribute to its mechanism of action • Further understand, in an immunohistochemical sub-study, the mechanisms of PRP that may account for its clinical efficacy • Identify the histological pathways that PRP may alter to exert its effects; using these results and those from the PRP biological component sub-study, inform future targeted manipulation of PRP properties to maximise its efficacy in tendon healing	At baseline (sub-study 1) • Blood sample for PRP component analysis (cell count, relevant tendon active growth factors, concentrations and platelet activation); all PRP group participants • Whole blood analysis for cell count and concentrations; all participants. At 6 weeks post treatment (sub-study 2): • A needle biopsy of the healing Achilles tendon under ultrasound guidance during an outpatient visit for immunohistochemistry analysis (*n* = 16)

### Eligibility criteria

Inclusion and exclusion criteria for the PATH-2 trial are presented in Table 2.

**Table 2:** PATH-2 inclusion and exclusion criteria

Inclusion criteria	Exclusion criteria
• Acute Achilles tendon rupture • 18+ years of age • Presenting and receiving treatment within 12 days after injury • Referred to non-operative treatment • Ambulatory prior to injury without the use of walking aids or assistance • Willing to give informed consent and able to comply with all study requirements	• Achilles tendon injuries at the insertion to the calcaneum or at the musculo-tendinous junction • Previous major tendon or ankle injury or deformity to either lower leg • History of diabetes mellitus • Known platelet abnormality or haematological disorder • Current use of systemic cortisone or treated with anticoagulant • Evidence of lower limb gangrene/ulcers or peripheral vascular disease • History of hepatic or renal impairment or dialysis • Pregnant or breast feeding • Having received radiation or chemotherapy within the last 3 months • With inadequate venous access for drawing blood • Any other significant disease or disorder which, in the opinion of the Investigator, may either put the participant at risk or influence study results

### Intervention groups

Participants are randomly assigned (1:1) to one of two groups by the Oxford Clinical Trials Research Unit (OCTRU) online randomisation service:

- **Treatment:** local anaesthetic injection to skin, followed by PRP injection to the tendon rupture gap;
- **Control:** local anaesthetic injection to skin, followed by needle insertion into tendon rupture gap.

### Sample size

A total number of 214 patients (107 per arm) will provide 90% power to detect a standardised difference of 0.5 in the LSI measured at 24 weeks post treatment, with 5% (two-sided) significance and allowing 20% losses to follow-up. This is based on data from the non-surgical arm of a previous study [4], where a clinically important difference of 10% with a standard deviation (SD) of 20 was observed. This sample size will also provide 90% power and 5% (two-sided) significance to detect a standardised effect size of 0.5 in the Achilles Tendon Rupture Score (ATRS; patient reported outcome) between the two intervention groups, based on a difference of 11 and an SD of 21.4.

However, this sample calculation was inflated as a result of a pre-specified blinded check of the assumptions for the primary outcome variability, which took place approximately halfway through recruitment using the overall population data (total sample, rather than intervention groups separately), as recommended by the Data and Safety Monitoring Committee (DSMC). As of June 2017, approximately half of the patients had reached their primary end-point and recruitment was still ongoing. Using cleaned and validated data from 75 patients, the observed SD was 24. Based on this observed SD of 24 (being aware that this could go up or down with the addition of further patient data), the sample size was recalculated.

Therefore, based on a minimum of 80% power, and allowing for 20% loss to follow-up, a sample size of 226 patients would be required to identify a clinically important difference of 10% with an SD of 24. The DSMC advised us during this process, and it was decided that the recruitment should overshoot to a minimum of 230 patients (to account for further minimal fluctuations on sample size assumptions) as a feasible target in the remaining timelines of the trial. This approach was supported by the Trial Steering Committee (TSC).

### Recruitment

Patients are identified in the outpatient trauma/orthopaedic clinic. Information about the study is given to suitable patients who are willing to participate, as well as an invitation to further discussion with a member of the research team so they have the opportunity to ask questions. After gaining appropriate informed consent, baseline data is collected prior to undergoing the injection treatment.

### Initial and current randomisation scheme

After informed consent and baseline measurement have been completed, participants are randomly assigned to either PRP injection or imitation injection via a central computer-based allocation randomisation system provided by OCTRU, in a 1:1 allocation ratio, using undisclosed variable permuted block sizes with stratification by recruiting site and age group (< 55, ≥ 55 years).

- Until 16 April 2016, using variable blocks of undisclosed bock size stratified by study site. At this time, 43 patients were randomised.
- From 17 April 2016, using minimisation, stratified using study site and age group, with a probabilistic element of 0.8 being included to ensure balance at the end of the study [5].

The reason for changing the original randomisation scheme was that, during a routine monitoring of the randomisation system, it was discovered that the online system was incorrectly taking information from the age group field prior to it being populated. This led to the age strata not being considered and a slight imbalance of treatment allocations in the different age group strata.

To allow randomisation recovering from this imbalance, the randomisation system was updated to include minimisation (using site and age group entered manually to the system).

### Blinding

Participants should remain blind to allocation throughout the study. The study staff involved in treatment delivery are aware of treatment allocation due to the nature of the intervention. Blinded assessors unaware of treatment allocation will carry out study follow-up (including the HRET at 24 weeks). When any hospital notes are updated relating to treatment or General Practitioner letters dictated, it is recorded that an injection was delivered as per the random allocation assigned by the PATH-2 study without specifying the type of injection.

Participants are invited to participate in a secondary 24-month extended follow-up and therefore are not informed of their allocation after the 24-week primary endpoint follow-up. After the extended follow-up is complete, participants may be informed of their treatment allocation by post, text or email if a request is made. The James et al. [6] and Bang et al. [7] indices, designed to assess whether participants were successfully blinded to the intervention they received, will also be calculated and reported with respective 95% confidence intervals.

### Data collection, quality control and data validation

Baseline data is collected from participants and site personnel via paper case report forms (CRFs), which are then returned to the central trial office by post using a Freepost address (pre-paid). In some cases, CRFs may be collected during a site visit/face-to-face meeting with a member of the local team. Data collection at the 4, 7 and 13 week follow-up points is done by telephone call or during a face-to-face outpatient appointment with responses recorded directly onto the CRFs. Where necessary, a member of the central research team in Oxford carries out telephone follow-up. A postal option of data collection may be used as necessary.

Blood and needle biopsy samples sent for analysis are anonymised at source and only identified using the unique study number and participant initials. Blood samples are stored at the Centre for Translational Inflammation Research at the Birmingham University Research Laboratory, and will be disposed of at the end of the study. Needle biopsy samples for those participants taking part in sub-study 2 (*n* = 16) are stored in the Oxford Musculoskeletal Biobank. Any data provided from the blood sample analysis or biopsy samples analysis are entered into the study database in Oxford. Data will be transferred using appropriate password protected and/or encrypted files.

The HRET data is collected from the participant during a face-to-face visit at 24 weeks. Movement data during the HRET are recorded via the linear encoder linked to a study-dedicated laptop, then transferred to the study-dedicated database in Oxford. A study-specific version of the MuscleLab software (Ergotest Innovation AS, Norway) is used to run and record the HRET data. Since the encoder is a very sensitive device, it records even minimal movements that might not correspond to actual heel rises. To dismiss potential measurement errors, where a participant provides consent, a video is made of the ankle/leg movements associated with the HRET. The video file contains no identifying details; only the unique study number is used. The file is sent to the central coordinating team in Oxford where two members of the study team blinded to treatment allocation independently review them, discounting any invalid repetition included in the HRET data (e.g. minimal movements that do not consist in actual heel rises, but that are captured by the assessment device). To ensure validity of data, intra-class correlation coefficients will be calculated for the two revised measurements before consensus on the number of valid heel rise repetitions is achieved.

The central study office receives the CRFs and the coordinating team carries out appropriate data quality and validation checks. The data is entered into a study-dedicated database developed and maintained by OCTRU using OpenClinica software. To identify manual entry errors, a 10% double entry check of follow-up questionnaires is carried out at regular intervals during the data collection phase of the study. No data will be considered spurious in the analysis since all data will be checked and cleaned before analysis.

### Specification of statistical packages

All analysis will be carried out using appropriate validated statistical software such as STATA, SAS, SPLUS or R statistical software. The relevant package and version number will be recorded in the Statistical report.

### Interim analysis and stopping rules

There were no formal interim analyses of the outcomes planned for PATH-2. However, the DSMC has checked the sample size assumptions, as detailed above, and the funder carried out an assessment of study feasibility after the 10th month following the start of recruitment. The feasibility criteria considered are presented in Table 3.

**Table 3:** Trial feasibility criteria as per the funder request

Criteria	Target
Number of sites open to recruitment	7
Ratio of patients consented/eligible	≥ 20%
Number of participants randomised	≥ 50
Compliance with intervention	Subjective, percentage of non-compliers to be considered
Loss to follow-up	≤ 30%
Safety	Subjective, all adverse events and serious adverse events considered

These criteria were met and discussed with the independent DSMC, and a recommendation for the continuation of the study was made to the independent TSC. The TSC confirmed the final decision and reported to the funder. The DSMC did not request any further interim analyses.

### Descriptive analysis of participant flow

**Fig. 1:**
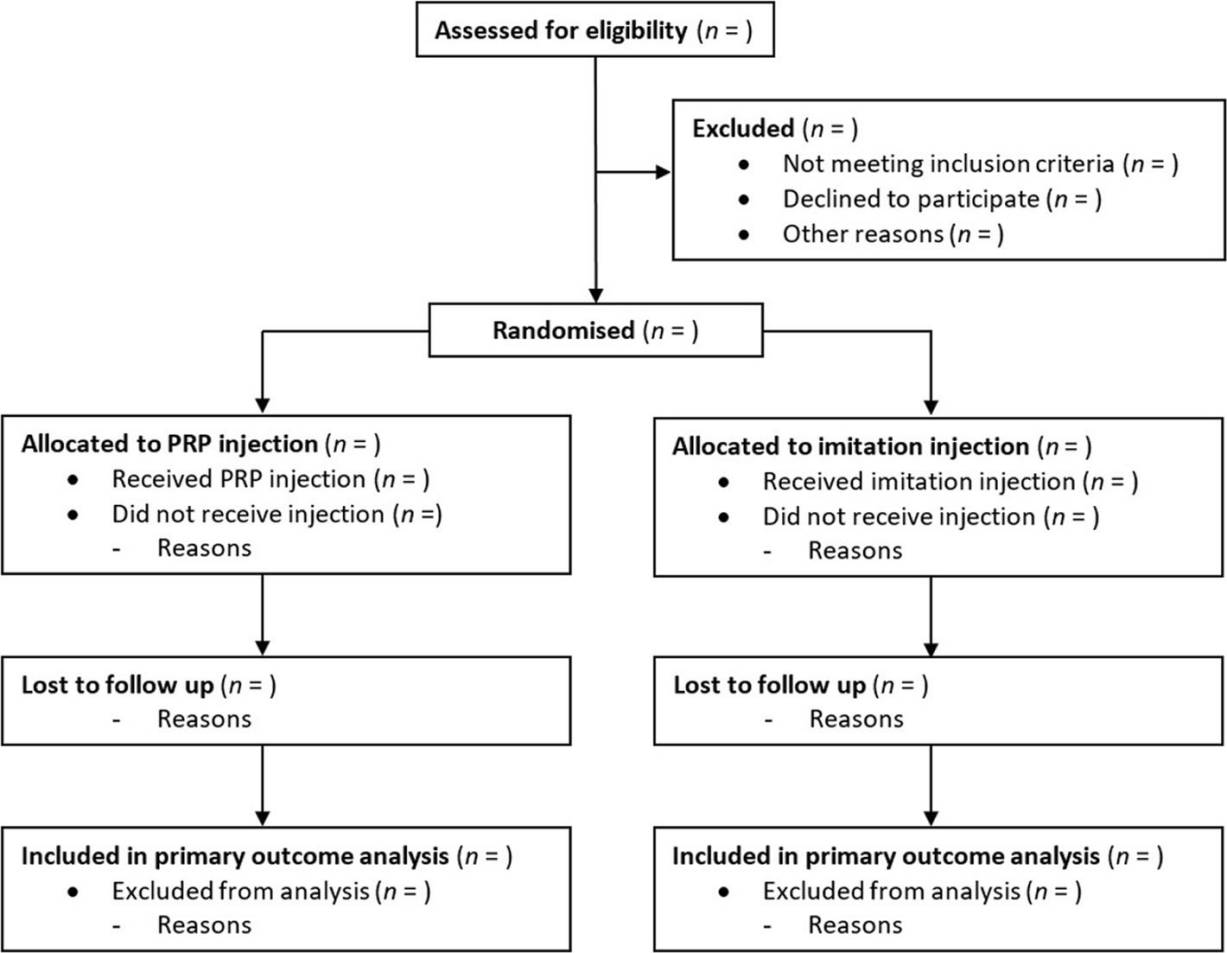
CONSORT flow diagram of participants in the trial

A summary of participants through the trial will be presented for each group. The number of participants through each stage of the trial will be provided in a flow diagram as suggested by CONSORT (Fig. 1). Protocol violations/deviations and information relating to the screening data, including the number of ineligible patients randomised, together with reasons, will be also reported.

### Baseline comparability of randomised groups

Numbers (with percentages) for binary or categorical variables and means (with standard deviations) or medians (with lower and upper quartiles) for continuous variables will be presented; there will be no tests of statistical significance nor confidence intervals for differences between randomised groups on any baseline variable. Additional file 1: Tables S1–S8 give the complete list of baseline variables.

### Comparison of losses to follow-up

The numbers (with percentages) of losses to follow-up (defaulters and withdrawals) over the study time points, and for each analysis endpoint, will be reported and compared between the PRP and imitation injection groups. To ensure that there are no differential losses between the groups, this will be tested using absolute risk differences (with 95% confidence interval) and a χ^2^ test. Deaths and their causes will be reported separately.

### Quality assurance and compliance with intervention

Intervention involves a single injection of PRP after blood sample collection and PRP preparation, or the introduction of a dry needle, into the tendon tissue [3]. Quality assurance will be undertaken using the times recorded regarding key processes related to the intervention preparation and delivery. A summary of the treatment received will be provided, including information on the timeframes for PRP preparation and analysis and grade of the healthcare professional delivering the treatment. Compliance with treatment and any protocol deviations prior to or during the intervention procedure will be reported totally and separately by treatment arms with reasons for not receiving the assigned injection. A χ^2^ test (or equivalent) will be used to examine whether compliance differs significantly between intervention groups.

### Blinded analysis

A blinded analysis of data (not separated by treatment arm) will be undertaken prior to the final data lock in order to clarify whether continuous variables are normally distributed and to finalise the identification of the participants who will be excluded from the per-protocol (PP) analysis. This analysis may also be used to identify key prognostic variables to be included in the adjusted analysis. The Statistical Analysis Plan will be updated before the final analysis of the trial, in case of any changes at this stage. These changes will be justified in the final Study Report.

### Definition of populations for analysis

- **Intention-to-treat (ITT) analysis:** inclusion of all randomised participants who will be analysed in the groups to which they were allocated. For the primary outcome, analysis will include a modified ITT population defined as all randomised ITT participants with available LSI data (i.e. at least one valid rep for each lower limb after HRET data validation by the blinded reviewers of the assessment video files).
- **Per-protocol (PP) analysis:** participants analysed in the group of the treatment they actually received. Patients who had major protocol violations/deviation will be excluded from the PP population. Patients who receive ‘poor quality’ PRP injection (e.g. PRP with concentrations of platelets lower than in their whole blood) will be excluded. The definition of PP analysis population will be finalised during a blinded analysis of the data (not separated by treatment) prior to the primary analysis time point and justified in the final Study Report.
- **Safety analysis:** All participants who received the intervention that they were allocated to, as defined in the protocol.

### Analyses to address the primary aim

For the primary outcome, analysis will be a performed on a modified ITT basis as defined above, comparing intervention (PRP injection) against control (Imitation injection). A two-sided *p* value of 0.05 (5% significance level) will be used to indicate statistical significance. Exact *p* values will be presented up to three decimal places. Multivariate linear regression, using the LSI as a continuous dependent variable, treatment as the main independent variable and the stratification factors (centre and age group) as additional independent variables, will be used to investigate the adjusted effect of PRP on ATR recovery. Supplementary analyses will repeat the primary analysis also adjusting for other prognostic factors such as sex, body mass index, smoking status and use of antiplatelet medication. The primary analysis used to assess the treatment effect will be the fully adjusted analysis based on the modified ITT population, with the other analyses being used to ensure robustness of results.

If data on the primary outcome is not normally distributed, the first approach will be data transformation (such as logarithm, exponential or other type of transformation). If normality cannot be achieved by data transformation, a non-parametric statistical test (e.g. Wilcoxon–Mann–Whitney test for testing whether the population medians of the two groups are the same) will be used. In this case, no adjustment will be made.

### Missing data

There are two main categories of missing data for the HRET assessment, namely (1) missing data for specific repetitions of heel rise (values of displacement missing or recorded as zero by the software) and (2) missing data for entire assessments (for one or both legs; derived from missing appointments, refusal or inability to perform the test appropriately, and missing data files). The first approach to handling missing data will be to impute the average (mean or median values, depending on the data distribution; checked for the total population during the blinded analysis) concentric displacement (upwards movement) for missing measurements due to the unlikely event of technical device failure during the conduct of an assessment (first group of missing data). Multiple imputation by chained equations [8] may be used to estimate the LSI for those patients with entire files compromised or unavailable for any reason (second group of missing data) and for the prognostic variables, in case of substantial amount of missing data (e.g. > 20% for a single variable). If multiple imputation is used, then this will be regarded as a sensitivity analysis to assess the robustness of the results. In this case, multiple imputation by chained equations will comprise the same variables included in the fully adjusted model used to investigate the effect of the intervention on the primary outcome (i.e. the stratification and other prognostic factors such as sex, body mass index, smoking status and use of antiplatelet medication), plus an indicator of treatment group and the outcome. This process creates a series of complete datasets (observed + imputed data) in which the analyses described in this document will be performed individually. The regression parameter estimates plus corresponding standard errors obtained from the analyses of each imputed dataset will then be combined using Rubin’s Rules [9]. Where missing data imputation is used, imputed values will also be verified using the validation checks described above to ensure the missing values have been imputed within the limits of the data.

### Pre-specified subgroup analysis

No formal subgroup comparative analysis is planned. However, treatment effects within subgroups, assessed using interaction between treatment and the subgroup, will be presented using forest plots to visually examine whether the effect of PRP injection compared to imitation differs based on the stratification factors (age group and centre) and other prognostic variables such as sex, body mass index categories, smoking status, PRP quality (assessed in terms of platelet concentration and activation) and pain levels during the first 2 weeks after treatment (as measured by the visual analogue scale diaries).

### Sensitivity analysis

A sensitivity analysis will be performed on a PP basis to examine robustness of conclusions and to assess different assumptions about departures from randomised policies. The PP population is as defined above and will be finalised during the blinded analysis. Sensitivity analysis excluding potential outliers or extremely unexpected observations (e.g. patients with a value of LSI much higher than 100%, meaning that the injured leg performed much better than the uninjured one in the HRET) will be conducted to check the robustness of the main findings.

### Analysis to address secondary aims

The primary analysis for all secondary outcomes will be performed on an ITT basis, comparing intervention (PRP injection) against control (Imitation injection). For the analyses of the secondary outcomes a *p* value of 0.05 (5% significance level) will be used to indicate statistical significance and treatment effects reported with 95% confidence intervals. Exact *p* values will be presented up to three decimal places.

Linear mixed effects regression models will be used to allow the data collected at all follow-up time-points to be considered and adjusting for pre-injury and baseline scores where applicable. This is a robust procedure capable of dealing with some missing values, either due to missed visits or to a patient leaving the study prematurely. Time elapsed from the intervention to the outcome measurement will be included in the models as a random effect factor, considering that not all patients will have their follow-up assessments at exactly the same time. These analyses will be adjusted for the stratification factors and, if applicable, for the same prognostic factors included in the adjusted analysis of the primary outcome. Mean differences and 95% confidence intervals will also be reported. If normality is not observed for any of the continuous secondary outcomes, the first approach will be data transformation. If normality cannot be achieved by data transformation, the same strategy adopted for the primary outcome will be used.

Self-reported pain in the first 2 weeks after randomisation will also be represented graphically using information obtained from the pain diary. Supplementary analyses will include using area under the curve summary statistics calculated from parameters estimated with linear mixed models, to provide an overall estimate of recovery over time [10]. Pain for each treatment group will also be explored using the pain section of the ATRS score collected at 4, 7, 13 and 24 weeks.

### Pre-specified subgroups and sensitivity analyses for secondary outcomes

No formal subgroup comparative analyses will be undertaken for secondary outcomes. However, results for key secondary outcome (ATRS) will be presented using forest plots as described above for the primary outcome. In the particular case of the ATRS, the study sample provides 90% power to detect a difference of 11 points with an SD of 21.4 (standardised effect size of 0.5) at 5% (two-sided) significance level. Sensitivity analysis will be performed on a PP basis to examine robustness of conclusions based on the results observed for the ATRS. This will be to investigate different assumptions about departures from randomised policies, as well as to check the validity of multiple imputation assumptions where applicable.

### Supplementary analysis: 24-month participant reported data

Patient-Specific Functional Scale, visual analogue scale and ATRS data will be collected at 24 months after injury by post or telephone call to permit an exploratory analysis of any effects of PRP beyond 24 weeks. A difference in speed of healing, if evident, will be seen when the tendon is in the recovery phase; the primary outcome measure at 6 months after injury is timed to capture this data. However, restoring the final level of function, sport and work occurs over a longer period following ATR, with an overall recovery time of 2 or more years post injury [1]. If PRP affects the quality of the repaired tendon, we would expect to see this at 2 years post injury. Academic/employment status and physical activity-related data will also be collected at 24 months and exploratory analysis will be performed. These supplementary analyses will be published separately from the main study results.

### Health economics and cost effectiveness

No health economics or cost-effectiveness analysis is planned for PATH-2.

### Harms

To the best of our knowledge there have been no serious adverse events (SAEs) related to using PRP reported in the literature. Unforeseeable events related to the study treatments will be reported as an SAE if they take place within 24 weeks of receiving trial treatment/imitation and fulfil the SAE criteria. Other adverse events related to the trial interventions and condition may be reported by site staff or by patients. Following completion of follow-up, duplicate adverse event reports will be identified, such that each adverse event is recorded no more than once for each patient. The number (and percentage) of patients experiencing each type of event will be presented for each treatment arm, categorised as per the protocol (Table 4). The safety analysis for PATH-2 will be performed on the Safety population as defined above. SAEs will be monitored at each follow-up point. A comparison of SAEs between the PRP and imitation injection groups will be assessed by examination of 95% confidence intervals for the difference in incidence. An overall category for any SAE will also be compared.

**Table 4:** Serious adverse events and adverse events

Serious adverse events An untoward medical occurrence that: • Results in death • Is life-threatening • Requires in-patient hospitalisation or prolongation of existing hospitalisation • Results in persistent or significant disability/incapacity • Leads to a congenital anomaly/birth defect • Other important medical events	
Adverse events (related to study treatment or condition that do not require specific time-critical reporting but may be collected as part of standard data collection in the PATH-2 trial) Foreseeable adverse events • Bruising and discomfort at the venesection site • Mild discomfort or minor bleeding from ATR site following injection • Technical complications of the lower leg casting and splinting • Consequences of depending on walking aids • Syncopal (fainting) episode associated with venesection or tendon injection • Discomfort at ATR site during rehabilitation • Swelling or bruising of the lower leg and foot • Deep vein thrombosis in a lower limb • Re-rupture of the treated Achilles tendon (including any surgery on the Achilles tendon treated in the study) Unforeseeable adverse events • Serious infection of ATR injection site • Skin breakdown or ulceration of treated lower leg other than ‘plaster sores’ • Severe pain requiring more than simple analgesia beyond 10 days after injection Other adverse events not pre-specified but deemed related to treatment or condition will also be recorded and reported	

### Additional exploratory analysis not specified prior to receiving data

Any analyses not specified in this Statistical Analysis Plan will be exploratory in nature and a significance level of 0.01 will be used to declare statistical significance; 99% confidence intervals will be presented.

### Patient and public involvement

The PATH-2 TSC includes a patient and public involvement representative who advises on patients’ priorities, experiences and preferences. This includes, but is not restricted to, recruitment strategies, burden of intervention, relevance of outcomes and dissemination of results.

### Ethics and dissemination

The trial will be reported in accordance with the CONSORT statement [11] and its extensions relating to non-pharmacological studies [12, 13] and TIDieR (template for intervention description and replication) guidelines for intervention description and replication [14]. Further details on the ethical aspects of the trial and dissemination plan can be found in the protocol publication [3].

## Discussion

This manuscript was written based on the PATH-2 Protocol version 6.0 and SAP version 2.0. This Statistical Analysis Plan publication will avoid bias arising from prior knowledge of the study results. Any changes or deviations from the current Statistical Analysis Plan will be described and justified in the final report.

### Update on recruitment closure and follow-up

The PATH-2 trial recruited participants between July 2015 and September 2017. Follow-up for the primary analysis will be completed by March 2018 and the trial is due to report results in August 2018.

## Additional file

Additional file 1: **Table S1.** Baseline characteristics of participants according to intervention groups. **Table S2.** Stratification factors and sociodemographic characteristics of participants according to intervention groups. **Table S3.** Baseline patient reported outcomes by intervention groups. **Table S4.** Academic/employment-related characteristics of participants according to intervention groups. **Table S5.** Physical activity of participants before injury according to intervention groups. **Table S6.** Injury-related characteristics of participants by intervention groups. **Table S7.** Clinical characteristics of participants by intervention groups. **Table S8.** Medications that may influence platelet function taken by participants by intervention groups. **Table S9.** Complications reported by participants during the 24 weeks after injury and treatment. (PDF 1944 kb)

## Abbreviations

ATRS: Achilles Tendon Rupture Score
CRF: Case Report Form
DSMC: Data and Safety Monitoring Committee
HRET: Heel Rise Endurance Test
ITT: Intention-To-Treat
LSI: Limb Symmetry Index
NHS: National Health Service
OCTRU: Oxford Clinical Trials Research Unit
PP: Per Protocol
PRP: Platelet-rich Plasma
SAE: Serious Adverse Event
SD: Standard Deviation
TSC: Trial Steering Committee
