# Supplementary material for: Platelet-rich plasma in Achilles tendon healing 2 (PATH-2) trial: statistical analysis plan for a multicentre, double-blinded, parallel-group, placebo-controlled randomised clinical trial
Source: Trials. 2018 Aug 29;19:464. doi: 10.1186/s13063-018-2840-z (PMC6114254; doi:10.1186/s13063-018-2840-z)
Supplement: Supplementary file 1 — Table S1. Baseline characteristics of participants according to intervention groups. Table S2. Stratification factors and sociodemographic characteristics of participants according to intervention groups. Table S3. Baseline patient reported outcomes by intervention groups. Table S4. Academic/employment-related characteristics of participants according to intervention groups. Table S5. Physical activity of participants before injury according to intervention groups. Table S6. Injury-related characteristics of participants by intervention groups. Table S7. Clinical characteristics of participants by intervention groups. Table S8. Medications that may influence platelet function taken by participants by intervention groups. Table S9. Complications reported by participants during the 24 weeks after injury and treatment. (PDF 1944 kb) [file 13063_2018_2840_MOESM1_ESM.pdf]

Table S1. Baseline characteristics of participants according to intervention groups.

Baseline characteristics	PRP injection			Imitation			Total		
	n	Mean	SD	n	Mean	SD	n	Mean	SD
Height (m)	XXX	X.XX	X.XX	XXX	X.XX	X.XX	XXX	X.XX	X.XX
Weight (kg)	XXX	XX.X	X.XX	XXX	XX.X	X.XX	XXX	XX.X	X.XX
BMI (kg/m^2)	XXX	XX.X	X.XX	XXX	XX.X	X.XX	XXX	XX.X	X.XX
Age (years)	XXX	XX.X	X.XX	XXX	XX.X	X.XX	XXX	XX.X	X.XX
Alcohol consumption (units)	XXX	XX.X	X.XX	XXX	XX.X	X.XX	XXX	XX.X	X.XX
Days since injury	XXX	X.X	X.XX	XXX	X.X	X.XX	XXX	X.X	X.XX

Table S2. Stratification factors and socio-demographic characteristics of participants according to intervention groups.

Stratification factor	PRP injection		Imitation		Total	
	n	%	n	%	n	%
Centre						
John Radcliffe Hospital, Oxford	XXX	XX	XXX	XX	XXX	XX
Musgrove Park Hospital, Taunton	XXX	XX	XXX	XX	XXX	XX
Southmead Hospital, Bristol	XXX	XX	XXX	XX	XXX	XX
Llandough Hospital, Cardiff	XXX	XX	XXX	XX	XXX	XX
Royal London Hospital	XXX	XX	XXX	XX	XXX	XX
Leicester Royal Infirmary	XXX	XX	XXX	XX	XXX	XX
University Hospital Coventry	XXX	XX	XXX	XX	XXX	XX
Warrington Hospital	XXX	XX	XXX	XX	XXX	XX
Basildon University Hospital	XXX	XX	XXX	XX	XXX	XX
Royal Liverpool Hospital	XXX	XX	XXX	XX	XXX	XX
Peterborough City Hospital	XXX	XX	XXX	XX	XXX	XX
Morriston Hospital, Swansea	XXX	XX	XXX	XX	XXX	XX
University Hospital Aintree	XXX	XX	XXX	XX	XXX	XX
Wythenshawe Hospital, Manchester	XXX	XX	XXX	XX	XXX	XX
Northern General Hospital, Sheffield	XXX	XX	XXX	XX	XXX	XX
Royal Devon & Exeter Hospital	XXX	XX	XXX	XX	XXX	XX
Royal Surrey Hospital, Guildford	XXX	XX	XXX	XX	XXX	XX
Leighton Hospital, Crewe	XXX	XX	XXX	XX	XXX	XX
Age (years)						
< 55	XXX	XX	XXX	XX	XXX	XX
≥ 55	XXX	XX	XXX	XX	XXX	XX
Gender						
Female	XXX	XX	XXX	XX	XXX	XX
Male	XXX	XX	XXX	XX	XXX	XX
Lower limb injured						
Right	XXX	XX	XXX	XX	XXX	XX
Left	XXX	XX	XXX	XX	XXX	XX
Body mass index (kg/m^2)						
Underweight (< 18.5)	XXX	XX	XXX	XX	XXX	XX
Normal weight (18.5–24.99)	XXX	XX	XXX	XX	XXX	XX
Overweight (25–29.99)	XXX	XX	XXX	XX	XXX	XX
Obese (≥ 30)	XXX	XX	XXX	XX	XXX	XX
Smoking status						
Never smoked	XXX	XX	XXX	XX	XXX	XX
Ex-smoker	XXX	XX	XXX	XX	XXX	XX
Smoker	XXX	XX	XXX	XX	XXX	XX

Table S3. Baseline patient reported outcomes (PROMs) by intervention groups.

Patient reported outcome measures	PRP injection			Imitation			Total		
	n	Mean	SD	n	Mean	SD	n	Mean	SD
Pain VAS score (0-100)	XX	X.XX	X.XX	XX	X.XX	X.XX	XX	X.XX	X.XX
PSFS (0-10)	XX	X.XX	X.XX	XX	X.XX	X.XX	XX	X.XX	X.XX
ATR (0-100)	XX	X.XX	X.XX	XX	X.XX	X.XX	XX	X.XX	X.XX
Pain score from ATRS (0-10)	XX	X.XX	X.XX	XX	X.XX	X.XX	XX	X.XX	X.XX
SF-12 (physical score before injury)	XX	X.XX	X.XX	XX	X.XX	X.XX	XX	X.XX	X.XX
SF-12 (mental score before injury)	XX	X.XX	X.XX	XX	X.XX	X.XX	XX	X.XX	X.XX
SF-12 (physical score baseline)	XX	X.XX	X.XX	XX	X.XX	X.XX	XX	X.XX	X.XX
SF-12 (mental score baseline)	XX	X.XX	X.XX	XX	X.XX	X.XX	XX	X.XX	X.XX

Table S4. Academic/employment related characteristics of participants according to intervention groups.

Academic / employment status	PRP Injection		Imitation		Total	
	n	%	n	%	n	%
Academic status						
Full-time student	XXX	XX	XXX	XX	XXX	XX
Part-time student	XXX	XX	XXX	XX	XXX	XX
Not studying	XXX	XX	XXX	XX	XXX	XX
Employment status						
More than 40 hours / week	XXX	XX	XXX	XX	XXX	XX
25-40 hours / week	XXX	XX	XXX	XX	XXX	XX
10-25 hours / week	XXX	XX	XXX	XX	XXX	XX
Less than 10 hours / week	XXX	XX	XXX	XX	XXX	XX
Unemployed, looking for work	XXX	XX	XXX	XX	XXX	XX
At home / not looking for paid employment	XXX	XX	XXX	XX	XXX	XX
Unable to work due to illness or disability	XXX	XX	XXX	XX	XXX	XX
Fully retired (no paid work)	XXX	XX	XXX	XX	XXX	XX
Type of work						
Office based	XXX	XX	XXX	XX	XXX	XX
Shop work (or similar)	XXX	XX	XXX	XX	XXX	XX
Classroom (or equivalent)	XXX	XX	XXX	XX	XXX	XX
Physical outside work	XXX	XX	XXX	XX	XXX	XX
Physical indoor work	XXX	XX	XXX	XX	XXX	XX
Mainly traveling/on the road	XXX	XX	XXX	XX	XXX	XX
Other 1	XXX	XX	XXX	XX	XXX	XX
Other 2	XXX	XX	XXX	XX	XXX	XX
etc.	XXX	XX	XXX	XX	XXX	XX
Time spent on feet						
Most of the day	XXX	XX	XXX	XX	XXX	XX
More than 4 hours / day	XXX	XX	XXX	XX	XXX	XX
Less than 4 hours / day	XXX	XX	XXX	XX	XXX	XX
Not much time (mostly sitting)	XXX	XX	XXX	XX	XXX	XX
Time spent driving						
Most of the day	XXX	XX	XXX	XX	XXX	XX
More than 4 hours / day	XXX	XX	XXX	XX	XXX	XX
Less than 4 hours / day	XXX	XX	XXX	XX	XXX	XX
Just to/from work	XXX	XX	XXX	XX	XXX	XX
Do not drive	XXX	XX	XXX	XX	XXX	XX

Table S5. Physical activity of participants before injury according to intervention groups.

Physical activity	PRP injection		Imitation		Total	
	n	%	n	%	n	%
Fitness (Cycling, jogging/running, weight-training, aerobics, etc.)						
More than once / week	XXX	XX	XXX	XX	XXX	XX
Less than once / week	XXX	XX	XXX	XX	XXX	XX
Never	XXX	XX	XXX	XX	XXX	XX
Cycling						
More than once / week	XXX	XX	XXX	XX	XXX	XX
Less than once / week	XXX	XX	XXX	XX	XXX	XX
Never	XXX	XX	XXX	XX	XXX	XX
Jogging/running						
More than once / week	XXX	XX	XXX	XX	XXX	XX
Less than once / week	XXX	XX	XXX	XX	XXX	XX
Never	XXX	XX	XXX	XX	XXX	XX
Walking						
More than once / week	XXX	XX	XXX	XX	XXX	XX
Less than once / week	XXX	XX	XXX	XX	XXX	XX
Never	XXX	XX	XXX	XX	XXX	XX
Weight-training						
More than once / week	XXX	XX	XXX	XX	XXX	XX
Less than once / week	XXX	XX	XXX	XX	XXX	XX
Never	XXX	XX	XXX	XX	XXX	XX
Aerobics/keep-fit						
More than once / week	XXX	XX	XXX	XX	XXX	XX
Less than once / week	XXX	XX	XXX	XX	XXX	XX
Never	XXX	XX	XXX	XX	XXX	XX
Athletics						
More than once / week	XXX	XX	XXX	XX	XXX	XX
Less than once / week	XXX	XX	XXX	XX	XXX	XX
Never	XXX	XX	XXX	XX	XXX	XX
Ball sports (Football, rugby, hockey, netball)						
More than once / week	XXX	XX	XXX	XX	XXX	XX
Less than once / week	XXX	XX	XXX	XX	XXX	XX
Never	XXX	XX	XXX	XX	XXX	XX
Football						
More than once / week	XXX	XX	XXX	XX	XXX	XX
Less than once / week	XXX	XX	XXX	XX	XXX	XX
Never	XXX	XX	XXX	XX	XXX	XX
Rugby						
More than once / week	XXX	XX	XXX	XX	XXX	XX
Less than once / week	XXX	XX	XXX	XX	XXX	XX
Never	XXX	XX	XXX	XX	XXX	XX
Hockey						
More than once / week	XXX	XX	XXX	XX	XXX	XX
Less than once / week	XXX	XX	XXX	XX	XXX	XX
Never	XXX	XX	XXX	XX	XXX	XX
Netball						
More than once / week	XXX	XX	XXX	XX	XXX	XX
Less than once / week	XXX	XX	XXX	XX	XXX	XX
Never	XXX	XX	XXX	XX	XXX	XX

Table S5. Cont.

Physical Activity	PRP injection		Imitation		Total	
	n	%	n	%	n	%
Tennis						
More than once / week	XXX	XX	XXX	XX	XXX	XX
Less than once / week	XXX	XX	XXX	XX	XXX	XX
Never	XXX	XX	XXX	XX	XXX	XX
Squash						
More than once / week	XXX	XX	XXX	XX	XXX	XX
Less than once / week	XXX	XX	XXX	XX	XXX	XX
Never	XXX	XX	XXX	XX	XXX	XX
Badminton						
More than once / week	XXX	XX	XXX	XX	XXX	XX
Less than once / week	XXX	XX	XXX	XX	XXX	XX
Never	XXX	XX	XXX	XX	XXX	XX
Non-sporting activity (Heavy DIY, housework, gardening, etc.)						
More than once / week	XXX	XX	XXX	XX	XXX	XX
Less than once / week	XXX	XX	XXX	XX	XXX	XX
Never	XXX	XX	XXX	XX	XXX	XX
Heavy DIY, housework, gardening						
More than once / week	XXX	XX	XXX	XX	XXX	XX
Less than once / week	XXX	XX	XXX	XX	XXX	XX
Never	XXX	XX	XXX	XX	XXX	XX
Others (1, 2, 3, etc…)						
More than once / week	XXX	XX	XXX	XX	XXX	XX
Less than once / week	XXX	XX	XXX	XX	XXX	XX
Never	XXX	XX	XXX	XX	XXX	XX

Table S6. Injury related characteristics of participants by intervention groups.

Injury characteristics	PRP injection		Imitation		Total	
	n	%	n	%	n	%
Mechanism of injury						
During sports	XXX	XX	XXX	XX	XXX	XX
Heavy DIY, housework, gardening, etc.	XXX	XX	XXX	XX	XXX	XX
Other	XXX	XX	XXX	XX	XXX	XX
Activity during which sustained tendon rupture						
Cycling	XXX	XX	XXX	XX	XXX	XX
Jogging/running	XXX	XX	XXX	XX	XXX	XX
Walking	XXX	XX	XXX	XX	XXX	XX
Wight training	XXX	XX	XXX	XX	XXX	XX
Aerobics/keep fit	XXX	XX	XXX	XX	XXX	XX
Athletics	XXX	XX	XXX	XX	XXX	XX
Football	XXX	XX	XXX	XX	XXX	XX
Rugby	XXX	XX	XXX	XX	XXX	XX
Hockey	XXX	XX	XXX	XX	XXX	XX
Netball	XXX	XX	XXX	XX	XXX	XX
Tennis	XXX	XX	XXX	XX	XXX	XX
Squash	XXX	XX	XXX	XX	XXX	XX
Badminton	XXX	XX	XXX	XX	XXX	XX
Heavy DIY, housework, gardening	XXX	XX	XXX	XX	XXX	XX
Others (1, 2, 3, etc…)	XXX	XX	XXX	XX	XXX	XX

Table S7. Clinical characteristics of participants by intervention groups.

Clinical characteristics	PRP injection		Imitation		Total	
	n	%	n	%	n	%
Medical condition in addition to tendon rupture						
Heart disease	XXX	XX	XXX	XX	XXX	XX
Hypertension	XXX	XX	XXX	XX	XXX	XX
Asthma/COPD	XXX	XX	XXX	XX	XXX	XX
Parkinson's disease	XXX	XX	XXX	XX	XXX	XX
Epilepsy	XXX	XX	XXX	XX	XXX	XX
Liver disease	XXX	XX	XXX	XX	XXX	XX
Stroke/Mini-stroke (TIA)	XXX	XX	XXX	XX	XXX	XX
Peptic ulcer	XXX	XX	XXX	XX	XXX	XX
Cancer	XXX	XX	XXX	XX	XXX	XX
Deep Vein Thrombosis (DVT)/Pulmonary embolism (PE)	XXX	XX	XXX	XX	XXX	XX
Osteoarthritis	XXX	XX	XXX	XX	XXX	XX
Rheumatoid arthritis	XXX	XX	XXX	XX	XXX	XX
Others (1, 2, 3, etc…)	XXX	XX	XXX	XX	XXX	XX
Number of co-morbidities						
1	XXX	XX	XXX	XX	XXX	XX
2	XXX	XX	XXX	XX	XXX	XX
More than 2	XXX	XX	XXX	XX	XXX	XX
Taking medication for pain or inflammation before injury						
No	XXX	XX	XXX	XX	XXX	XX
Yes	XXX	XX	XXX	XX	XXX	XX

Table S8. Medications that may influence platelet function taken by participants by intervention groups.

Medication that could influence platelet function	PRP injection		Imitation		Total	
	n	%	n	%	n	%
Yes	XXX	XX	XXX	XX	XXX	XX
No	XXX	XX	XXX	XX	XXX	XX

Table S9. Complications reported by participants during the 24 weeks after injury and treatment.

	PRP injection		Imitation		Total	
	n	%	n	%	n	%
Serious Infection at injection site confirmed by laboratory analysis	XXX	XX	XXX	XX	XXX	XX
Infection at injection site confirmed by doctor	XXX	XX	XXX	XX	XXX	XX
Infection non-injection site	XXX	XX	XXX	XX	XXX	XX
Severe pain at injection site requiring more than simple pain relief	XXX	XX	XXX	XX	XXX	XX
DVT in lower limb	XXX	XX	XXX	XX	XXX	XX
Skin breakdown or ulceration in the treated leg	XXX	XX	XXX	XX	XXX	XX
Re-rupture of the Achilles tendon treated in the study	XXX	XX	XXX	XX	XXX	XX
Surgery on the Achilles tendon treated in the study	XXX	XX	XXX	XX	XXX	XX
Fainting due to blood sample collection	XXX	XX	XXX	XX	XXX	XX
Fainting due to study intervention delivery	XXX	XX	XXX	XX	XXX	XX
Frequent discomfort at injection site	XXX	XX	XXX	XX	XXX	XX
Discomfort at injection site only during rehabilitation exercises	XXX	XX	XXX	XX	XXX	XX
Swelling of lower leg or foot	XXX	XX	XXX	XX	XXX	XX
Plaster sores	XXX	XX	XXX	XX	XXX	XX
Technical complications with casting and splinting	XXX	XX	XXX	XX	XXX	XX
Consequences of depending on walking aids	XXX	XX	XXX	XX	XXX	XX
Other problem with cast, boot or splint	XXX	XX	XXX	XX	XXX	XX
Other problem	XXX	XX	XXX	XX	XXX	XX
Number of complications per participant						
Zero	XXX	XX	XXX	XX	XXX	XX
One	XXX	XX	XXX	XX	XXX	XX
Etc.	XXX	XX	XXX	XX	XXX	XX
SAEs						
List here (…)	XXX	XX	XXX	XX	XXX	XX

Notes: DVT: Deep vein thrombosis. n: number of participants reporting this type of complication one or more times during the course of the study. The number of complication events is not given, due to re-reporting by participants.
